# What Form of RSV Protection Do Women Prefer: Maternal Vaccination or Infant Immunisation? A Cross-Sectional Survey in Europe

**DOI:** 10.3390/vaccines14030238

**Published:** 2026-03-05

**Authors:** Diana Mendes, Malvin Kang, Amy Law

**Affiliations:** 1 Pfizer Ltd., Tadworth, Surrey KT20 7NS, UK; 2 Ipsos Pte Ltd., Singapore 189721, Singapore; 3 Pfizer Inc., New York, NY 10001, USA; amy.law@pfizer.com

**Keywords:** respiratory syncytial virus, maternal immunisation, maternal vaccination, infant immunisation, infant protection, preference

## Abstract

Background/Objectives: Respiratory syncytial virus (RSV) is a major cause of acute lower respiratory infections in infants/newborns, sometimes requiring hospitalisation. Two immunisation options are approved in Europe: maternal vaccination and infant immunisation. Success of the immunisation programme depends on uptake—however, understanding of women’s immunisation preferences remains limited. This study evaluated women’s knowledge of RSV, RSV immunisation intentions, and factors influencing immunisation decisions in Finland, France, Germany, Italy, Spain, and the UK. Methods: A cross-sectional survey was conducted between November and December 2024 with 740 women across the selected countries. Participants included women who were pregnant, trying to conceive, or previously pregnant within the year prior to survey. Participants were asked about their knowledge, intentions, and preferences regarding RSV immunisation. Results: Familiarity with RSV was lower than that for COVID-19 and influenza (45% vs. 86% and 85%). Among those pregnant or trying to conceive, 68% reported they were likely to initiate an RSV immunisation discussion with a healthcare provider and 21% reported they were unlikely to do so. The majority (76%) also reported they were likely to accept RSV vaccination if recommended by a healthcare provider. Overall, 52% preferred maternal vaccination over infant immunisation. Among the 82% of women who would consider RSV immunisation and had a preference for immunisation method, 68% favoured maternal vaccination. Conclusions: RSV immunisation acceptance among European women is high and a majority prefer maternal vaccination over infant immunisation. This indicates potential for RSV immunisation to achieve high uptake when women have access to immunisation methods aligned with their preferences.

## 1. Introduction

Respiratory syncytial virus (RSV) is a major cause of acute lower respiratory tract infections (LRTIs) in infants and newborns, leading to hospitalisations in severe cases [1,2]. Globally, RSV is responsible for an estimated 33 million RSV-associated LRTI episodes annually in children under five years of age, resulting in approximately 3.6 million hospitalisations and 100,000 deaths, the vast majority occurring in infants under six months of age [3]. Although mortality is highest in low- and middle-income countries, RSV remains a substantial cause of morbidity, hospitalisation, and healthcare utilisation in high-income regions, including Europe.

In the European Union, RSV is associated with about 245,000 annual hospital admissions for respiratory infection in children under five years of age, with 75% occurring in those under one year of age [1]. Severe RSV disease can lead to complications such as respiratory failure requiring intensive care, mechanical ventilation, and prolonged hospital stays [4]. In addition to acute morbidity, RSV infection in early life has been linked to long-term sequelae, including recurrent wheezing and an increased risk of developing asthma later in childhood [5]. Risk factors for severe RSV disease include prematurity, congenital heart disease, chronic lung disease, immunodeficiency, and young chronological age; however, most hospitalised infants are born at term and have no underlying medical conditions, underscoring the broad population-level impact of RSV [6,7]. RSV contributes substantially to seasonal surges in paediatric healthcare utilisation, underscoring the importance of implementing effective strategies to protect infants during their highest-risk period [1,8,9].

To protect infants during the peak seasonal period against RSV, two forms of RSV immunisation are currently approved in Europe: maternal vaccination and direct, passive immunisation of infants with long-acting monoclonal antibodies (mAbs). Maternal immunisation involves administration of a single dose of the RSVpreF vaccine to pregnant women within 24–36 weeks of gestation for the prevention of RSV-associated LRTI in infants via transplacental antibody transfer [10,11,12]. Passive immunisation of infants with the long-acting mAb nirsevimab provides direct protection against RSV for infants [13,14]. Both options have been approved by the European Medicines Agency (EMA) since 2022. As of October 2025, 23 countries recommend RSV immunisation, with 19 providing funded long-acting mAbs programmemes, 16 countries recommending universal long-acting mAbs for all infants, 3 focusing on high-risk cases, while 3 countries solely utilise maternal vaccination and 5 offer it as an alternative to long-acting mAbss [15,16,17,18,19,20]. France and the UK both offer fully funded universal long-acting mAbs for infants and maternal vaccination, allowing parents to choose between the two. Germany, Finland, and Spain have opted exclusively for fully funded universal infant programmes using long-acting mAbs, such as nirsevimab. However, Italy’s national strategy remains under discussion as of late 2025, with no funded universal program currently implemented at the national level [21].

With the availability of RSV immunisation options and RSV being identified by the World Health Organisation (WHO) as a global health priority [22], understanding how pregnant women and new mothers perceive and their choice between maternal vaccination and infant immunisation is critical for immunisation programme design and communication. Immunisation uptake is a key determinant of NIP success and is, in part, influenced by willingness to immunise and HCP recommendations [23,24,25].

Previous studies conducted in several European countries have highlighted a lack of RSV awareness and yet positive perceptions towards RSV immunisation among women. In addition, immunisation preferences have been found to lean toward maternal vaccination [23,24,25,26,27,28,29]. However, a comprehensive assessment of awareness, knowledge, intentions, preferences, and motivations across multiple European countries is lacking. In addition, studies to date have not assessed the influence of healthcare providers on European women’s decisions about RSV immunisation.

This study assessed the following: (1) women’s awareness and knowledge of RSV; (2) women’s intentions, preferences, and motivations regarding maternal/infant RSV immunisation; (3) factors influencing maternal/infant RSV immunisation decision-making across Europe.

## 2. Methods

### 2.1. Study Design and Variables

A cross-sectional, online survey in six European countries (Finland, France, Germany, Italy, Spain, and the UK) was conducted between 13 November and 10 December 2024. The questionnaire took approximately 10 min to complete and comprised single- and multiple-select items, including Likert scales. The questionnaire assessed women’s intentions, knowledge, preferences, and motivations regarding RSV immunisation (see Supplementary Materials).

The questionnaire was divided into three sections: The first collected respondent’s demographic characteristics and health-related information, including age, level of education, and pregnancy status. The second section assessed familiarity with respiratory illnesses and knowledge of risk factors associated with children’s risk of RSV infection. The third section gathered respondents’ self-reported likeliness to take certain actions, including initiating a conversation with a healthcare provider about RSV immunisation and accepting a recommendation for RSV vaccination.

### 2.2. Study Participants

Participants for this study were recruited from established online research panels managed by Dynata Global UK Ltd (London, UK). To address recruitment associated with pregnancy status, recruitment efforts focused on women aged 20–45 years. Nevertheless, all potential participants, regardless of gender, were presented with the same initial information and underwent identical screening criteria. Before gaining access to the questionnaire, participants were required to review an online informed consent form. This form detailed the study’s objectives, procedures, voluntary participation nature, and data confidentiality assurances. Only those who provided electronic consent were allowed to continue with the survey.

Across the six surveyed countries (United Kingdom, France, Germany, Italy, Spain, and Finland), a total of 1258 respondents participated in the survey (UK: n = 373; France: n = 210; Germany: n = 140; Italy: n = 236; Spain: n = 154; Finland: n = 145).

Eligible respondents were females aged 18 years or older who were pregnant (“currently pregnant”), were trying to conceive, or had been pregnant within the past 12 months at the time of the survey. No respondents were excluded at recruitment on the basis of general opposition to immunisation or affiliation with the healthcare sector.

### 2.3. Ethics

The study was conducted as part of syndicated market research and adhered to the guidelines of the Declaration of Helsinki. Participation was voluntary and uncoerced, with no incentives provided. Data were collected anonymously and handled in compliance with the General Data Protection Regulation (GDPR). The study, aiming to evaluate the knowledge and intentions regarding RSV immunisation, employed a non-interventional survey methodology typical of healthcare market research in compliance with the British Healthcare Business Intelligence Association (BHBIA) and European Pharmaceutical Market Research Association (EphMRA) guidelines. As the study involved fully anonymised data and did not constitute any interaction with survey respondents or the processing of personal data, a review by an institutional review board or ethics committee was not required. Furthermore, no adjustments were made to the informed consent form for women’s participation.

### 2.4. Data Analysis

Data was analysed descriptively. Multiple-choice survey question results are reported as the number and/or proportion of respondents selecting each option. Results of numerical survey questions are reported as means. Only responses from completed questionnaires were included in the analysis. SPSS (Version 28.0.1.1, IBM Corp., Armonk, NY, USA) was used for data tabulation and cleaning.

## 3. Results

### 3.1. Demographics

Out of 1250 respondents who began the questionnaire, 740 eligible women completed the survey (completion rate: 59%). The exclusions due to incomplete surveys are distributed as follows: UK (n = 166), France (n = 94), Germany (n = 40), Italy (n = 92), Spain (n = 28), and Finland (n = 98), totalling at 518.

The mean age of respondents was 33 years and the majority of respondents were in the 30–39 year age group (n = 385, 52%) (Table 1). At the time of the survey, 56% (n = 411) of respondents were pregnant, 30% (n = 223) were trying to conceive, and 14% (n = 106) had been pregnant within the previous 12 months. Most participants (n = 456, 62%) held a university degree, and 71% (n = 525) were parents. Many participants had been vaccinated recently: 55% (n = 407) for influenza, 31% (n = 229) for Tetanus/Diphtheria/Pertussis (whooping cough) (Tdap), and 47% (n = 348) for COVID-19 within the last 12 months.

### 3.2. Awareness and Knowledge of RSV

Self-reported familiarity with RSV (n = 333, 45%), was lower than for other respiratory diseases, including COVID-19 (n = 636, 86%), influenza (n = 629, 85%), pneumonia (n = 474, 64%), and bronchiolitis (n = 429, 58%) (Table 2). When asked to identify risk factors in RSV, no single risk factor was identified by more than 45% of respondents. The most commonly identified risk factors were weakened immune system (n = 296, 45%), daycare or sick-adult exposure (n = 260, 39%), and premature birth (n = 240, 36%). Zero risk factors (i.e., “I don’t know”) were identified by 16% (n = 106) of respondents (Table 2). Only 34% of respondents were able to identify age ≤ 6 months as a risk factor in RSV infection.

### 3.3. Likelihood to Initiate Conversations About RSV Immunisation

Among respondents currently pregnant or trying to conceive (n = 634), 68% (n = 431) indicated that they would be likely to initiate a discussion about RSV immunisation with a healthcare provider. However, 20% (n = 27) reported that they would be unlikely do so and 12% (n = 76) reported that they were neither likely nor unlikely to initiate such a conversation.

### 3.4. Likelihood to Accept Maternal RSV Vaccination

**Figure 1:**
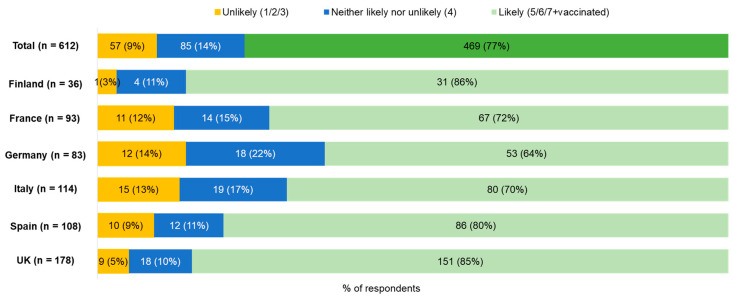
Likelihood to accept maternal RSV vaccination if recommended by a healthcare provider among respondents who were pregnant or trying to conceive (n = 612).

Among respondents who were pregnant or trying to conceive (n = 612), 77% (n = 469) reported that they would be likely to accept maternal RSV vaccination if it were recommended by a healthcare provider (i.e., rated 5–7 on a 7-point scale of likelihood, or were already vaccinated). However, 14% (n = 85) reported that they were unsure whether they would accept a recommendation for maternal vaccination (i.e., rated 4) and 9% (n = 57) indicated that they were vaccine hesitant (i.e., rated 1–3) (Figure 1).

### 3.5. Preferences for Method of Immunisation

**Figure 2:**
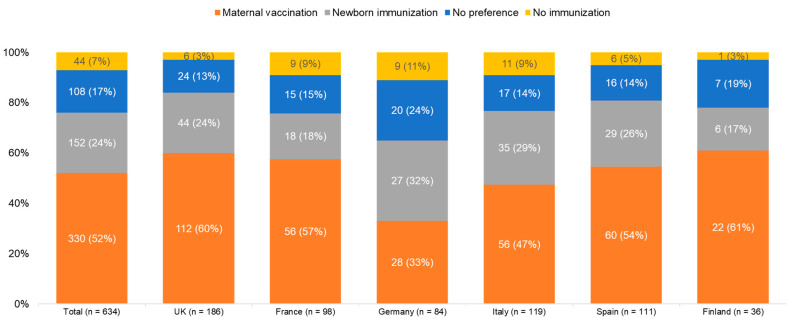
RSV immunisation method preference among respondents who were pregnant or trying to conceive (n = 634).

Regarding the method of immunisation, slightly over half of respondents (n = 330, 52%) who were pregnant or trying to conceive indicated that they would prefer maternal vaccination over infant immunisation. In comparison, 24% (n = 152) indicated that they would prefer infant immunisation over maternal vaccination, while 17% (n = 108) had no preference and 7% (n = 44) reported that they would not consider RSV immunisation. (Figure 2).

By aggregating the two preference categories, we observe that a significant majority of respondents, specifically 482 individuals (76%), who were either pregnant or attempting to conceive, displayed a preference for a certain method of immunisation. Among this group, a notable 68% (n = 330) favoured maternal vaccination, while the remaining 32% (n = 152) expressed a preference for infant immunisation.

### 3.6. Factors Influencing Acceptance of Maternal Vaccination During Pregnancy

Respondents who stated that they were likely to accept maternal RSV vaccination if recommended by a healthcare provider were asked to identify the factors influencing their willingness to be vaccinated. The most commonly reported factors were related to safety for mother and/or child (n = 377, 65%), efficacy of the vaccine (n = 353, 61%), immediate protection of the infant from birth (n = 230, 40%), and recommendation by a healthcare provider (n = 211, 37%) (Table 3).

### 3.7. Factors Influencing Hesitancy Around Maternal Vaccination During Pregnancy

Respondents who stated that they were unlikely to accept maternal RSV vaccination if recommended by a healthcare provider (n = 78) were likewise asked to identify their reasons. The most commonly reported reasons were safety concerns (n = 36, 47%), lack of knowledge (n = 24, 31%), and lack of recommendation by the respondent’s own healthcare provider (n = 17, 21%) (Table 4). However, only a minority of respondents indicated they were unlikely to accept maternal vaccination (n = 7, 9%).

## 4. Discussion

This study highlights substantial gaps in awareness and knowledge of RSV among women who are pregnant, trying to conceive, or recently pregnant across the surveyed countries. Moderate levels of intention to initiate conversations about RSV immunisation in this population were noted. Nonetheless, the results indicate a strong willingness to accept maternal vaccination when recommended by a healthcare provider and identify a preference for maternal vaccination over infant immunisation.

The limited baseline knowledge noted from this study was consistent with previous similar studies in several countries and often underestimated the severity of RSV infection in infants [26,27,30,31,32]. Similar patterns of preference for maternal vaccination over infant immunisation have also been reported in other studies, where expectant parents prioritised high vaccine efficacy, safety, and protection immediately from birth while perceiving maternal vaccination to be more convenient than infant immunisation [27,30,33,34,35].

Across these studies, willingness to accept maternal RSV vaccination is consistently shaped by perceptions of vaccine safety, expected effectiveness, duration of protection, knowledge of RSV, and the influence of healthcare provider recommendation [26,27,30,31,34].

Our findings of lower familiarity with RSV and lack of knowledge regarding RSV risk factors in infancy underscore the need for RSV education among pregnant women. These findings suggest that education regarding the safety and efficacy of maternal RSV vaccination is likely to influence women’s decisions about whether to get vaccinated. Patient education on RSV vaccine safety should consider the safety of both the mother and child, including long-term safety. Effective patient education that emphasises both maternal and neonatal safety, alongside highlighting protection available from birth when infants are most vulnerable, is essential.

Finally, HCPs should consider engaging patients about RSV protection early during pregnancy. This would ensure that pregnant women can make an informed decision for their preferred method of immunisation to protect the prospective infant, while allowing time for discussion and consideration.

Overall, these findings provide insights informing policymakers and healthcare providers in the development of targeted educational interventions. In particular, targeted patient education within this population may play a critical role in improving immunisation uptake and broadening infant protection against RSV disease.

### Strengths and Limitations

This study benefits from a large heterogeneous sample of women who were of the primary stakeholders for receiving RSV immunisation. This sample also provided broad geographic coverage across Europe. Nevertheless, these findings are also subject to several limitations. First, the results are based on self-reported responses to survey questions and therefore may be subject to various biases, such as social desirability bias and/or priming. Second, the sample was drawn from an online panel which may introduce selection bias. In addition, the study was designed as a descriptive assessment of awareness, attitudes, and stated intentions rather than as a hypothesis-driven or causal analysis, and study outcomes were not analysed by controlling for potential confounders and variation of the study sample. The sample was not designed or powered to support inferential statistical testing, multivariable modelling, or formal hypothesis comparisons. Accordingly, the absence of *p*-values, confidence intervals, or regression analyses reflects the intent of the study, as inclusion of such measures could imply inferential conclusions that extend beyond the scope of the dataset. Finally, the sample size for respondents who indicate they are unlikely to accept maternal RSV vaccination is small, thus limiting the ability to draw strong conclusions regarding reasons for vaccine hesitancy. Future studies with larger, more diverse samples and analytic designs are warranted to build upon these findings.

## 5. Conclusions

Our findings suggest low awareness and knowledge of RSV among pregnant women or women who were trying to conceive in Europe. Nonetheless, there is high acceptability of RSV immunisation among European women, with maternal vaccination being preferred over infant immunisation. This highlights the critical role of healthcare professionals in having proactive, early conversations with prospective mothers about RSV and the importance of policymakers in providing access to immunisation options that align with their preferences.

## Figures and Tables

**Table 1:** Respondent demographics by country (n = 740).

	Total (n = 740)	Finland (n = 47)	France (n = 116)	Germany (n = 100)	Italy (n = 144)	Spain (n = 126)	UK (n = 207)
Age							
Mean	33	30	34	32	35	34	31
18–29	222 (30%)	20 (43%)	32 (28%)	31 (31%)	24 (17%)	27 (21%)	87 (42%)
30–39	385 (52%)	26 (55%)	55 (47%)	56 (56%)	84 (58%)	69 (55%)	89 (43%)
40+	133 (18%)	1 (2%)	29 (25%)	13 (13%)	36 (25%)	30 (24%)	31 (15%)
Pregnancy Status							
Currently pregnant	411 (56%)	20 (43%)	60 (52%)	60 (60%)	60 (42%)	61 (48%)	150 (73%)
Trying to conceive	223 (30%)	16 (34%)	38 (33%)	24 (24%)	59 (41%)	50 (40%)	36 (17%)
Pregnant in past 12 months	106 (14%)	11 (23%)	18 (16%)	16 (16%)	25 (17%)	15 (12%)	21 (10%)
Parental Status							
Parent	525 (71%)	34 (72%)	87 (75%)	67 (67%)	98 (68%)	91 (72%)	151 (73%)
Non-parent	215 (29%)	13 (28%)	29 (25%)	33 (33%)	46 (32%)	35 (28%)	56 (27%)
Vaccines received in the last 12 months							
Influenza	407 (55%)	24 (51%)	51 (44%)	58 (58%)	79 (55%)	78 (62%)	128 (62%)
Tdap	229 (31%)	15 (32%)	35 (30%)	34 (34%)	37 (26%)	37 (29%)	77 (37%)
COVID-19	348 (47%)	16 (34%)	53 (46%)	44 (44%)	60 (42%)	71 (56%)	118 (57%)
Education Status							
University Graduate	456 (62%)	29 (62%)	77 (66%)	50 (50%)	91 (63%)	69 (55%)	153 (74%)

**Table 2:** Familiarity with respiratory diseases and risk factors for RSV (n = 740).

Country	Number of respondents, n (%)						
	Total	Finland	France	Germany	Italy	Spain	UK
Self-Reported Familiarity (n = 740)							
RSV	333 (45%)	25 (53%)	26 (22%)	56 (56%)	58 (40%)	54 (43%)	118 (57%)
COVID-19	636 (86%)	41 (87%)	90 (78%)	85 (85%)	132 (92%)	101 (80%)	193 (93%)
Influenza	629 (85%)	40 (85%)	88 (76%)	86 (86%)	131 (91%)	98 (78%)	190 (92%)
Pneumonia	474 (64%)	33 (71%)	58 (50%)	71 (71%)	76 (53%)	79 (63%)	153 (74%)
Bronchiolitis	429 (58%)	15 (32%)	71 (61%)	60 (60%)	104 (72%)	81 (64%)	124 (60%)
Knowledge on RSV risk factors (n = 660)							
Weakened immune system	296 (45%)	20 (50%)	44 (42%)	35 (38%)	46 (38%)	46 (43%)	115 (58%)
Premature birth (before 32 weeks)	240 (36%)	13 (33%)	40 (38%)	36 (39%)	31 (26%)	37 (35%)	93 (47%)
Age 6 months or younger	223 (34%)	20 (50%)	26 (25%)	34 (37%)	37 (31%)	27 (25%)	69 (35%)
Children with chronic lung disease	195 (30%)	12 (30%)	40 (38%)	25 (27%)	25 (21%)	27 (25%)	71 (36%)
Children with heart disease (present at birth)	195 (30%)	12 (30%)	35 (34%)	19 (21%)	36 (30%)	28 (26%)	71 (36%)
Children who have neuromuscular disorders	149 (23%)	10 (25%)	24 (23%)	12 (13%)	22 (18%)	25 (24%)	63 (32%)
Children exposed in daycare or to sick adults	260 (39%)	23 (58%)	32 (31%)	31 (34%)	42 (35%)	42 (40%)	75 (38%)
Child with a genetic disorder	128 (19%)	7 (18%)	21 (20%)	17 (18%)	18 (15%)	21 (20%)	50 (25%)
I don’t know	106 (16%)	3 (8%)	23 (22%)	14 (15%)	23 (19%)	19 (18%)	28 (14%)

**Table 3:** Factors influencing maternal RSV vaccination acceptance among respondents likely to accept maternal RSV vaccination if recommended by a healthcare professional (n = 578).

Factor	Number of Respondents, n (%) by Country						
	Total (n = 578)	Finland (n =35)	France (n = 86)	Germany (n = 73)	Italy (n = 105)	Spain (n = 102)	UK (n = 177)
Safety *	*377 (65%)*	*28 (80%)*	*53 (62%)*	*41 (56%)*	*69 (66%)*	*61 (60%)*	*119 (67%)*
Safe for baby	261 (45%)	22 (63%)	38 (44%)	25 (34%)	46 (44%)	38 (37%)	87 (49%)
Safe during pregnancy	252 (44%)	19 (54%)	30 (35%)	28 (38%)	44 (42%)	44 (43%)	89 (50%)
Safe for me	210 (36%)	15 (43%)	33 (38%)	20 (27%)	38 (36%)	36 (35%)	69 (39%)
Efficacy **	*353 (61%)*	*26 (74%)*	*55 (64%)*	*42 (58%)*	*53 (50%)*	*54 (53%)*	*119 (67%)*
Vaccine protects baby immediately at birth	230 (40%)	18 (51%)	29 (34%)	27 (37%)	35 (33%)	42 (41%)	76 (43%)
Prevents serious complications/symptoms	230 (40%)	18 (51%)	34 (40%)	24 (33%)	36 (34%)	38 (37%)	78 (44%)
Prevents hospitalizations	173 (30%)	17 (49%)	23 (27%)	17 (23%)	24 (23%)	29 (28%)	53 (30%)
Prevents transmission to others	148 (26%)	12 (34%)	19 (22%)	22 (30%)	20 (19%)	20 (20%)	51 (29%)
Others							
Recommendation from healthcare provider	211 (37%)	15 (43%)	22 (26%)	18 (25%)	41 (39%)	36 (35%)	90 (51%)
Multi-year protection	145 (25%)	12 (34%)	22 (26%)	17 (23%)	22 (21%)	27 (26%)	35 (20%)
Advice from government bodies	137 (24%)	9 (26%)	10 (12%)	27 (37%)	27 (26%)	16 (16%)	44 (25%)
Advice from pregnancy support groups	124 (22%)	8 (23%)	12 (14%)	23 (32%)	17 (16%)	17 (17%)	48 (27%)
Insurance covers cost	108 (19%)	—	16 (19%)	21 (29%)	13 (12%)	12 (12%)	37 (21%)
Believe baby will be at risk for RSV	100 (17%)	7 (20%)	14 (16%)	16 (22%)	11 (10%)	16 (16%)	35 (20%)
Advice from friends/family	87 (15%)	6 (17%)	10 (12%)	11 (15%)	7 (7%)	18 (18%)	37 (21%)
Nothing would influence decision	22 (4%)	—	5 (6%)	3 (4%)	2 (2%)	3 (3%)	7 (4%)

* Includes “Safe for baby”, Safe during pregnancy” and “Safe for me”. ** Includes “Vaccine protects baby immediately at birth”, “Prevents serious complications/symptoms”, “Prevents hospitalizations”, and “Prevents transmission to others”.

**Table 4:** Reasons for being unlikely to accept maternal RSV vaccination among respondents who were unlikely to accept maternal RSV vaccination (n = 78).

Country	Number of Respondents, n (%)						
	Total (n = 78)	Finland (n = 1)	France (n = 17)	Germany (n = 12)	Italy (n = 19)	Spain (n = 12)	UK (n = 17)
Safety and Side Effects *	*37 (47%)*	*-*	*6 (35%)*	*9 (75%)*	*12 (63%)*	*3 (25%)*	*6 (35%)*
I think the vaccine could harm my baby	21 (27%)	-	4 (24%)	6 (50%)	8 (42%)	1 (8%)	2 (12%)
I think the vaccine could harm me	15 (19%)	-	1 (6%)	4 (33%)	6 (32%)	1 (8%)	3 (18%)
I think the vaccine could put my pregnancy at risk	21 (27%)	-	3 (18%)	6 (50%)	6 (32%)	2 (17%)	3 (18%)
I am worried about the long-term side effects with the vaccine	29 (37%)	1 (100%)	4 (24%)	4 (33%)	5 (26%)	3 (25%)	2 (12%)
I am worried about the short-term side effects with the vaccine	14 (18%)	-	2 (12%)	4 (33%)	4 (21%)	1 (8%)	3 (18%)
Lack of knowledge **	*24 (31%)*	*-*	*7 (41%)*	*4 (33%)*	*7 (37%)*	*2 (17%)*	*5 (29%)*
I do not know enough about RSV vaccine(s)	21 (27%)	-	6 (35%)	4 (33%)	4 (21%)	2 (17%)	5 (29%)
I do not know enough about RSV as an illness	14 (18%)	-	2 (12%)	2 (17%)	5 (26%)	-	-
Concerns on Efficacy ***	*13 (17%)*	*-*	*1 (6%)*	*1 (8%)*	*5 (26%)*	*3 (25%)*	*3 (18%)*
I am concerned that the vaccine(s) will not offer protection for a long enough period of time	6 (8%)	-	1 (6%)	1 (8%)	1 (5%)	-	2 (12%)
I am concerned about how well the vaccine works	10 (13%)	-	1 (6%)	1 (8%)	4 (21%)	3 (25%)	1 (6%)
Others							
I don’t believe my baby is at risk for RSV	9 (11%)	-	2 (12%)	2 (17%)	1 (5%)	-	-
It has not been recommended by my healthcare provider (HCP)/pharmacist	17 (21%)	-	3 (18%)	4 (33%)	4 (21%)	2 (17%)	3 (18%)
I am focused on getting other vaccines right now	7 (9%)	-	1 (6%)	1 (8%)	-	1 (8%)	2 (12%)
I am tired of receiving vaccinations	7 (8%)	-	1 (6%)	1 (8%)	2 (11%)	-	-
I am against vaccinations in general	7 (9%)	-	3 (18%)	1 (8%)	1 (5%)	-	1 (6%)
I am concerned about cost / affordability of a vaccine	8 (10%)	-	1 (6%)	1 (8%)	1 (5%)	2 (17%)	2 (12%)
I am concerned because the vaccine(s) are new	15 (19%)	-	5 (29%)	-	3 (16%)	1 (8%)	4 (24%)

* Includes “I think the vaccine could harm my baby”, “I think the vaccine could harm me”, “I think the vaccine could put my pregnancy at risk”, “I am worried about the long-term side effects with the vaccine”, and “I am worried about the short-term side effects with the vaccine”. ** Includes “I do not know enough about RSV vaccine(s)” and “I do not know enough about RSV as an illness”. *** Includes “I am concerned that the vaccine(s) will not offer protection for a long enough period of time” and “I am concerned about how well the vaccine works”.

## Data Availability

The data presented in this study are available upon request from the corresponding author.

## Supplementary Materials

The following supporting information can be downloaded at: https://www.mdpi.com/article/10.3390/vaccines14030238/s1, which contains the survey questionnaire used for this study.
