# Supplementary material for: What Form of RSV Protection Do Women Prefer: Maternal Vaccination or Infant Immunisation? A Cross-Sectional Survey in Europe
Source: Vaccines (Basel). 2026 Mar 5;14(3):238. doi: 10.3390/vaccines14030238 (PMC13030599; doi:10.3390/vaccines14030238)
Supplement: Supplementary file 1 [file vaccines-14-00238-s001.zip › vaccines-4112758-supplementary.pdf]

Supplementary Information: Survey Questionnaire

Welcome to our study. This syndicated survey is being conducted by Ipsos Healthcare, an independent market research agency. The results of the study will be purchased by pharmaceutical companies, who use the information to understand the management of the disease and to monitor the use of their and their competitor's products. One or more of these pharmaceutical companies directly commissioned the inclusion of a limited number of proprietary questions within the survey.

The purpose of this research is to understand awareness and perceptions of RSV (respiratory syncytial virus) and the likelihood to receive an RSV vaccine as and when they are made available. This research is not promotional or intended to influence vaccination behaviors.

We will capture your thoughts via a 20-minute online questionnaire.

As a member of the Market Research Society (MRS), Ipsos Healthcare is bound by the MRS Code of Conduct and all applicable laws protecting your personal data and responses. The study is conducted in compliance with MRS/ ESOMAR/ EphMRA [UK ONLY:/ British Healthcare Business Intelligence Association] guidelines. Any information you provide us with will be treated as confidential, it will be combined with feedback from others like yourself. You will remain anonymous unless you give permission to be identified. Your information will only be used for market research and will not be passed to any other organization without your permission.

In this survey we would like to ask some questions that may be perceived as sensitive such as gender identification and ethnicity. Providing information in response to these questions is entirely voluntary and you may withdraw your consent at any time. The answers that you provide will be used only for market research analysis purposes.

1. Are you happy to proceed with the survey on this basis?
   - Yes
   - No [TERMINATE]

Section 1: Demographics & Background

Q1. Age
Please record your month and year of birth in the space provided.
Month: ______ Year: ______

Q2. Pregnancy Status
Which best describes your current situation?
☐ Currently pregnant

☐ Trying to get pregnant
☐ Pregnant in the last 12 months

Q3. Do you currently have any children under the age of 18 years?
☐ Yes
☐ No

Q4. Have you received any of the following vaccines in the last 12 months (select all that apply):
☐ Influenza (flu)
☐ COVID-19
☐ Tdap
☐ Other: ________

Q5. What is the highest level of education that you have achieved?
☐ Degree = Postgraduate degree, Undergraduate degree
☐ No Degree = Higher education below degree level, High school or equivalent, No formal educational qualifications

Section 2: Knowledge & Awareness

Q6. How familiar are you with the following illnesses?
RSV: ☐ Not at all ☐ Slightly ☐ Moderately ☐ Very ☐ Extremely
Influenza: ☐ Not at all ☐ Slightly ☐ Moderately ☐ Very ☐ Extremely
COVID-19: ☐ Not at all ☐ Slightly ☐ Moderately ☐ Very ☐ Extremely

Q7. Which of the following increases a child's risk of RSV? (select all that apply)
☐ Weakened immune system
☐ Premature birth (before 32 weeks)
☐ Age 6 months or younger
☐ Children with chronic lung disease
☐ Children with chronic heart disease (present at birth)
☐ Children who have neuromuscular disorders
☐ Children exposed in daycare or to sick adults
☐ Child with a genetic disorder
☐ I don't know

Section 3: Vaccination Intent & Preferences

Q8. If your doctor or healthcare provider recommends that you receive an RSV vaccine during your pregnancy between:

- **France:** 32–36 weeks
- **UK:** 28 weeks up to baby's birth
- **All other markets:** 24–36 weeks

to help protect your child at birth, how likely are you to get an RSV vaccine as recommended?

Please assume that you would easily be able to get the vaccine if you wanted it, and that the cost would be fully or mostly covered.

(Please rate on a scale of 1 to 7, where 1 = Definitely will not get an RSV vaccine and 7= Definitely will get an RSV vaccine or I have already received an RSV vaccine during my current pregnancy)
1 ☐ 2 ☐ 3 ☐ 4 ☐ 5 ☐ 6 ☐ 7 ☐

Q9. How likely are you to initiate a conversation with your doctor / healthcare provider (HCP) about receiving an RSV vaccine in the future? (For myself during pregnancy to protect my child(ren) at birth)?

(Please rate on a scale of 1 to 7, where 1 = Definitely will not initiate a discussion with my HCP about an RSV vaccine and 7 = Definitely will initiate a discussion with my HCP about an RSV vaccine)
1 ☐ 2 ☐ 3 ☐ 4 ☐ 5 ☐ 6 ☐ 7 ☐

Q10. If your doctor or healthcare provider allows you to make the choice to get vaccinated against RSV during pregnancy between:

- **France:** 32–36 weeks
- **UK:** 28 weeks up to baby's birth
- **All other markets:** 24–36 weeks

to protect your child at birth OR your child can be vaccinated/immunized against RSV after birth, which would you prefer?

☐ I would prefer to be vaccinated during my pregnancy to protect my child at birth
☐ I would prefer to vaccinate/immunize my child after birth
☐ I would not have a preference
☐ I would not consider getting vaccinated during pregnancy or vaccinating/immunizing my child

Q11. What factors below are/were most influential in driving your decision to get an RSV vaccine during your pregnancy? (select all that apply)

☐ Recommendation/advice from my healthcare provider (e.g., doctor, nurse, etc.)
☐ My insurance covers the cost of the vaccine

☐ I believe my baby will be at risk for RSV
☐ The vaccine will protect my baby from RSV immediately at birth
☐ The vaccine helps prevent serious complications/symptoms of RSV in babies
☐ The vaccine helps prevent hospitalizations due to RSV in babies
☐ The vaccine helps prevent transmission of RSV to others
☐ The vaccine is safe for my baby
☐ The vaccine is safe for me
☐ The vaccine is safe for my pregnancy
☐ The vaccine offers multi-year protection
☐ Recommendation/advice from pregnancy support groups
☐ Recommendation/advice from friends and family
☐ Recommendation/advice from governmental bodies
☐ Other

Q12. What are your **top reason(s)** for <u>not</u> intending to receive an RSV vaccine/being unsure about receiving an RSV vaccine during your pregnancy to help protect your child(ren) at birth against RSV?
☐ I think the vaccine could harm my baby
☐ I think the vaccine could harm me
☐ I think the vaccine could put my pregnancy at risk
☐ I don't believe my baby is at risk for RSV
☐ It has not been recommended by my healthcare provider (HCP)/pharmacist
☐ I am worried about the long-term side effects with the vaccine
☐ I am worried about the short-term side effects with the vaccine
☐ I am focused on getting other vaccines right now
☐ I do not know enough about RSV vaccine(s)
☐ I do not know enough about RSV as an illness
☐ I am tired of receiving vaccinations
☐ I am against vaccinations in general
☐ I am concerned about cost / affordability of a vaccine
☐ I am concerned because the vaccine(s) are new
☐ I am concerned that the vaccine(s) will not offer protection for a long enough period of time
☐ I am concerned about how well the vaccine works
☐ Other
